# CGRP: the immune system’s double agent – context-dependent roles in inflammation, resolution and cancer

**DOI:** 10.3389/fimmu.2026.1841366

**Published:** 2026-07-08

**Authors:** Mushref Bakri Assas

**Affiliations:** 1 Faculty of Applied Medical Sciences, Department of Medical Laboratory Technology, Immunology Group, King Abdul Aziz University, Jeddah, Saudi Arabia; 2 EcoHealth Unit, King Fahd Medical Research Centre, King Abdulaziz University, Jeddah, Saudi Arabia

**Keywords:** autoimmunity, cancer, CGRP - calcitonin gene-related peptide, migraine, neuroimmunology, tumour

## Abstract

Calcitonin gene-related peptide (CGRP) is traditionally recognized as a key mediator of nociception and vasodilation. However, it also functions as a context-dependent immune “double agent,” capable of both restraining inflammation and driving pathological responses. Here we discuss CGRP multifaceted roles in innate and adaptive immunity, inflammation, resolution, infection, autoimmunity, and cancer. In the innate immune response, CGRP predominantly exerts anti-inflammatory and tolerogenic effects on macrophages, mast cells, neutrophils, ILC2s, and NK cells, yet can promote pro-inflammatory signalling in acute high-DAMP (damage associated molecular patterns) settings. In adaptive immunity, it generally suppresses Th1 responses and induces CD8^+^ T cell exhaustion in chronic conditions, while paradoxically enhancing Th1 differentiation during acute viral infections. Aberrant TRPV1-CGRP signalling exacerbates IL-23/IL-17-driven pathology in psoriasis and experimental autoimmune encephalomyelitis, whereas CGRP depletion in severe systemic infections such as sepsis impairs neuro-immune protection. In the tumour microenvironment, tumours hijack sensory nerves to release CGRP, suppressing anti-tumour immunity, and directly stimulating cancer cell growth. This review highlights the potential of repurposing clinically approved anti-CGRP monoclonal antibodies—currently used for migraine—for conditions where elevated or dysregulated CGRP signalling drives pathology, while emphasizing the need for context-aware targeting to preserve physiological homeostasis.

## Introduction

The nervous and immune systems are highly coordinated to detect environmental threats, defend against pathogens, and to maintain physiological homeostasis (1, 2). Sensory nociceptors, form a critical interface in this bidirectional crosstalk; upon activation by exogenous/endogenous stimuli, inflammatory mediators, or immune signals, they release neuropeptides directly into local tissues (1, 3). Calcitonin gene-related peptide (CGRP), a 37-amino acid neuropeptide produced primarily by unmyelinated C-fibres is a master orchestrator in an ever expanding neuro-immune axis (2).

While historically recognized for its potent vasodilatory properties and its involvement in pain transmission pathways such as in migraine (4), CGRP now actively shapes systemic and localized immune responses (5). While initially thought to be limited at the level of the protein by manipulating cytokine levels, direction and function potency (6), today CGRP exerts context-dependent immunomodulatory effects across both innate and adaptive immunity, generally driving a regulatory or immunosuppressive phenotype, but not always (7–10). Indeed, the immense power of the CGRP signalling axis is most evident when it becomes dysregulated in pathological states. In chronic inflammatory conditions such as psoriasis and inflammatory bowel disease (IBD), the activation of the Transient receptor potential vanilloid 1 (TRPV1)-CGRP nociceptive axis actively drives neurogenic inflammation and drives local immune pathology (11, 12).

While the majority of mechanistic insights into CGRP’s immunomodulatory actions originate from murine genetic knockout models, experimental injury/infection systems, and *in vitro* studies, an increasing body of human evidence supports key observations. The calcitonin receptor-like receptor/receptor activity-modifying protein CLR/RAMP1 expression and CGRP-mediated signalling have been confirmed in human immune cells, while clinical data from migraine patients and tumour cohorts provide correlative support for its roles in neuro-immune crosstalk and immune evasion (8, 9, 13–17). In this review, most mechanistic insights i.e. signalling, cell specific effect, knockout phenotypes and inflammatory models (acute/chronic) come from murine models or *in vitro* systems. Human data are mostly correlative (receptor expression, clinical observation in migraine studies and data from cancer cohorts and tumour studies. Human evidence remains largely correlative, consisting primarily of receptor expression data, clinical observations from migraine studies, and findings from cancer cohorts and tumour samples.

This review examines CGRP’s emerging roles of in immunology, detailing its specific regulatory effects on distinct immune cell, and its paradoxical roles in autoimmunity and oncology. Ultimately, we highlight the translational promise of leveraging clinically approved CGRP receptor antagonists—a current breakthrough in migraine management —as a strategy to target pathological neuro-immune complications and restore robust immune surveillance in complex diseases.

## CGRP signalling: CLR/RAMP1 the specialized receptor complex

CGRP mediates its biological effects through a unique heterodimeric G-protein-coupled receptor (GPCR) complex. This complex is made of 1) the calcitonin receptor-like receptor CLR, that serves as the ligand-binding unit, and 2) a receptor activity-modifying protein, primarily RAMP1, which is essential for ligand specificity and expression at the cell surface (18, 19). While CGRP can also bind the CLR/RAMP3 complex (the primary adrenomedullin receptor), its highest affinity remains for RAMP1 (20–22). Of note, this receptor complex is not restricted to CGRP and adrenomedullin, in fact, amylin binds to CLR/RAMP1/2/3 illustrating receptor plasticity and multilayer functionality. This receptor complex is abundantly found on many immune and barrier cells facilitating in part CGRP immune-related properties (13–16).

Conventionally, CGRP was thought to signal exclusively from the plasma membrane. However, recent paradigm-shifting studies using enhanced bystander BRET (ebBRET) and NanoBiT approaches have revealed that CGRP initiates signalling at the level of the plasma membrane, but the CLR/RAMP1 complex is rapidly internalized into early endosomes. Once inside the cell, CGRP coupling preferentially activates stimulatory G protein alpha subunits (Gαs proteins), which stimulate adenylyl cyclase to generate cyclic adenosine monophosphate (cAMP) (23), subsequently activating Protein Kinase A (PKA) and the cAMP response element-binding protein (CREB) (24–26). Depending on the cellular context, CGRP can also engage alternative pathways, such as the p38/HSP27 mitogen-activated protein kinase (MAPK) cascade (27). Although many of these signalling mechanisms, including endosomal internalization and pathway bias, were elucidated primarily in cell lines and murine systems, CLR/RAMP1 expression and cAMP/PKA activation have been confirmed on human immune and barrier cells (13–16).

## Positioning CGRP as the immune system’s leading double agent

It is reasonable to predict that CGRP exerts a greater impact on the immune response than any other sensory neuropeptide (28). While studies cited in this review detail its mechanistic influences, CGRP is particularly well positioned—due to its anatomical distribution, abundance, and pharmacokinetics—to play multifaceted roles in both innate and adaptive immunity (2). C-fibres extensively innervate the epithelium as well as primary and secondary immune organs, lying in close proximity to a wide array of structural and immune cells, both within the epithelium and at luminal surfaces (29). Furthermore, previous studies have shown that CGRP is secreted from C-fibres in substantially higher quantities than other sensory neuropeptides such as substance P and VIP, although release remains tissue- and stimulus-dependent (30). In the trigeminal and dorsal root ganglia, CGRP mRNA is expressed at higher levels than substance P mRNA, a pattern observed in nearly all nociceptive neurons (31). In addition, CGRP exhibits a relatively longer duration of action in tissues compared with most sensory neuropeptides (plasma half-life approximately 7 minutes), which enhances its capacity to modulate multiple physiological events (32). This higher neuronal prevalence and release capacity, combined with prolonged local action, enable CGRP to exert broader and more sustained immunomodulatory effects compared with other neuropeptides. Such positioning is consistent with its established interactions with diverse immune cells, including mast cells, dendritic cells, and macrophages, across multiple tissues. These anatomical and pharmacokinetic features are highly conserved between rodents and humans.

The following sections examine these context-dependent roles in detail, consistently framed through the lens of CGRP as the immune system’s double agent.

## CGRP as the immune system’s double agent: key determinants of its divergent effects

CGRP functions as the quintessential “double agent” of the neuro-immune axis, capable of promoting or restraining immune responses depending on specific contextual factors. For example, in acute high-damage associated molecular pattern (DAMP) environments such as early spinal cord injury, nerve-derived CGRP can bias macrophages toward a pro-inflammatory M1 phenotype, whereas in the subacute/chronic resolution phase it strongly promotes an anti-inflammatory, tissue-repairing M2 phenotype via cAMP/PKA signalling (24, 29). Similarly, CGRP drives Th1 differentiation and antiviral immunity during acute viral infections through RAMP3 on CD4^+^ T cells (22), but induces CD8^+^ T cell exhaustion in chronic tumour microenvironments via RAMP1 and the epigenetic regulator Prdm12 (33).

This contextual plasticity unifies the findings across innate immunity, adaptive immunity, autoimmunity, infection, and cancer presented in this review, reinforcing CGRP central role as a context-dependent modulator rather than a uniformly pro- or anti-inflammatory mediator (34). Its apparently paradoxical actions are thought to be determined by: (1) disease stage (acute versus chronic or resolution phase), (2) tissue and cellular microenvironment, (3) receptor composition (CLR/RAMP1 versus CLR/RAMP3 or other RAMPs), (4) cellular target, (5) ligand source (nerve-derived versus tumour- or immune cell-derived), and (6) inflammatory intensity and duration (high-DAMP acute settings versus low-grade chronic inflammation) (2, 32). However, to truly understand why CGRP behaves so unpredictably, we have to look closely at its physical constraints as a neurotransmitter—a perspective often overlooked when comparing it to traditional cytokines. While cytokines are built for long-distance broadcasting, traveling through the bloodstream at low concentrations to affect distant organs, neurotransmitters, in general, operate on a much more intimate scale. They are released locally, meaning their true functional power is concentrated right at the nerve terminal or perivascular space (31, 35). Because of this localized design, the local concentration of CGRP acts as a crucial biophysical switch. Under normal ‘resting conditions’ CGRP is found at approximately 20–100 pg/ml (or higher locally) near sensory nerves and drops to as low as single digit pg/ml levels in systemic circulation (36). Interestingly, during pathologies like a migraine attack, these systemic levels surge dramatically in relevant compartments such as jugular venous blood (36, 37). With prior knowledge of immune involvement in such pathologies (38–40). This massive variance suggests that immune cells react differently based on the sheer volume of CGRP hitting them, a phenomenon regulated by receptor affinity and local enzymatic cleanup. At the molecular level, CLR changes its shape and behaviour based on the RAMP protein it pairs with. When bound to RAMP1, it forms the classic CGRP receptor, which has a very high, sub-nanomolar affinity for the peptide (41, 42). Furthermore, when CLR pairs with RAMP3, it creates the Adrenomedullin 2 (AM2) receptor, which requires roughly 50 to 100 times more CGRP (or shows substantially lower affinity) to fully activate (41, 42). This disparity creates a natural threshold system. At a baseline level, CGRP only activates the highly sensitive RAMP1 complex, leaving RAMP3 relatively quiet, yet during a neurogenic surge or major tissue trauma, the local area is flooded. This deluge saturates RAMP1 and spills over to activate the lower-affinity RAMP3 complexes. Because RAMP3 carries a unique PSD-95 (Postsynaptic density protein 95) Dlg1 (Drosophila disc large tumour suppressor) ZO-1 (Zona occludens-1, a tight junction protein) (PDZ)-binding domain on its tail, it internalizes and signals (and recycles) differently than RAMP1, effectively re-routing the cell’s internal programming from standard maintenance to a completely different immune response (42). This local build-up isn’t just about how much CGRP the nerves pump out; it is also heavily gated by membrane-bound enzymes like neprilysin (NEP) and insulin-degrading enzyme (IDE) (43). In healthy tissue, these enzymes act as a highly efficient localized cleanup crew, chopping up CGRP to keep it localized and preventing it from drifting too far. However, when acute tissue damage or heavy oxidative stress hits, these enzymes can become overwhelmed or downregulated. Consequently, CGRP escapes degradation, allowing local concentrations to rocket to levels that immune cells would never encounter in a resting state. On the flip side, the immunosuppressive environment of a tumour can selectively manipulate these pathways (or related neuroimmune signalling), creating pockets of high CGRP that promote CD8^+^ T cell exhaustion and a non-responsive state (7, 9). How this local abundance interacts with disease timelines, receptor layout, and tissue environments in real-time remains a wide-open frontier. Cracking this concentration-dependent code will be key to safely and effectively targeting the neuro-immune axis in future therapies.

## CGRP modulation of innate immunity

CGRP serves as a critical innate immunomodulator, generally driving an anti-inflammatory or tolerogenic phenotype. Within the innate immune compartment, CGRP signalling actively restrains acute inflammation. Its effects on macrophage plasticity are highly spatiotemporally regulated. In models of spinal cord injury (SCI), acute exposure to CGRP alongside high concentrations of DAMPs can bias signalling toward a pro-inflammatory M1 response (24, 44). However, during the subacute and chronic phases of injury, CGRP signalling reliably facilitates a functional shift toward an alternatively activated, tissue-repairing M2 macrophage phenotype via the cAMP/PKA pathway, promoting the clearance of debris and the secretion of IL-10 (24, 45, 46). Furthermore, CGRP inhibits mast cell degranulation (16) preventing histamine and prostaglandin release, limits neutrophil recruitment and bactericidal activity (47, 48). CLR and RAMP1 heterodimeric complex on macrophages activates a robust intracellular signalling cascade (25, 41, 49–53). This process is primarily mediated by stimulatory Gs proteins that activate adenylate cyclase, leading to increased intracellular cAMP levels and the subsequent activation of PKA (50, 53). Alternative cascades involve the mitogen-activated protein kinase/Extracellular signal-Regulated Kinase (MAPK/ERK) and Phosphoinositide 3-kinase/Protein kinase B (PI3K/Akt) pathways, which further contribute to the fine-tuning of macrophage activity and neurogenic inflammation (24, 54, 55). This signalling generally skews macrophages away from a M1 state toward an anti-inflammatory M2 phenotype (24, 49, 56). This phenotypic shift is characterized by the upregulation of markers such as CD206 and Arginase-1 (Arg-1), while pro-inflammatory markers like CD86 and inducible nitric oxide synthase (iNOS) are reversed or downregulated (24, 53, 56). In the cornea, signalling can also promote the selective apoptosis of M1 macrophages while driving the remaining population toward a pro-repair state (53). Macrophage activation is inhibited through the restriction of migration, a reduction in phagocytic efficiency, and the suppressed secretion of pro-inflammatory cytokines such as TNF-α, IL-6, and IL-1β (57). Inflammatory responses are further attenuated by the inhibition of the NF-κB pathway and the suppression of the NLRP3 inflammasome (49, 56, 58). Reparative functions are enhanced via the cAMP/TSP-1 signalling axis, which promotes macrophage efferocytosis—the clearance of apoptotic cells—thereby resolving inflammation and promoting tissue repair (53). A specialized CGRP related neuro-immune-metabolic axis exists where mitochondrial respiration and ATP synthesis are rapidly and effectively constrained during macrophage activation (58). This metabolic control is achieved through the transcriptional downregulation of multiple mitochondrial complex subunits, which limits the macrophages’ capacity to drive downstream processes like excessive osteoclastogenesis during bone repair. The functional output of these interactions is highly context-dependent and governed by signalling bias, where the dynamic recruitment of effectors like Gq or β-arrestin alters the response (24). For instance, in the acute phase of spinal cord injury, a high-density milieu of DAMPs may synergize with or override typical signalling to transiently bias macrophages toward a pro-inflammatory output (24).

Furthermore, CGRP forms a crucial negative feedback loop regulating type 2 innate lymphoid cells (ILC2s). ILC2s uniquely express both CGRP and its receptor, RAMP1 (20). Upon stimulation with epithelial alarmins like IL-33, endogenous CGRP suppresses ILC2 proliferation and inhibits IL-13 production (20). IL-13 promotes mucus production by epithelial cells as well as driving the ‘epithelial escalator’ part of the ‘weep and sweep’ responses in the gut. Mice lacking Ramp1 exhibit exacerbated, runaway allergic airway inflammation and profound eosinophilia, highlighting CGRP’s role as a physiological brake on mucosal immunity (20). CGRP roles on ILC2 results in forming a feedback loop that prevents runaway mucosal allergic inflammation, and potentially hampering other ILC2 functions i.e. hypersensitivity type 1, Type 2 immune cytokine production. etc. One of the earliest findings was CGRP inhibitory roles on NK cell functions (59). CGRP exerts inhibitory effects on NK cells by increasing the intracellular cyclic AMP concentration. In pancreatic ductal adenocarcinoma (PDAC), CGRP indirectly suppresses NK cells. CGRP binds to RAMP1 on cancer-associated fibroblasts (CAFs), completely halting their secretion of IL-15, which starves NK cells of necessary recruitment and activation signals, detrimental to the immune response towards the tumour promoting tumour survival (60). Furthermore, the context-dependent regulation of NK-cell function extends well beyond CGRP-mediated suppression in the tumour microenvironment. Local tissue-derived signals play a critical role in shaping NK-cell effector responses, particularly in inflammatory barrier tissues, reinforcing the central theme that immune outcomes are highly dependent on the local microenvironment. For example, Baumdick et al. showed that epithelial cells in ulcerative colitis express the HLA-DP401 haplotype, which engages the activating receptor Natural Killer cell p44 protein (NKp44) on NK cells (61). This interaction drives pro-inflammatory cytokine production and promotes NKp44+ NK cell-mediated damage to the intestinal epithelium, illustrating how local MHC class II molecules can shift NK-cell activity toward tissue pathology. This study nicely complement our discussion of CGRP as a context-dependent immunomodulator. Just as CGRP can dampen NK-cell cytotoxicity in tumours, these findings highlight how specific receptor-ligand interactions in distinct tissue environments can profoundly influence NK-cell behaviour.

Importantly, while macrophage polarization, ILC2 regulation, and NK cell suppression have been mechanistically detailed in murine models of spinal cord injury and allergic inflammation, supportive evidence for CLR/RAMP1 expression and anti-inflammatory effects exists from human ex vivo studies on macrophages and mast cells (13, 15, 16, 53, 62).

**Table 1** captures the predominantly anti-inflammatory/tolerogenic/suppressive role of CGRP on innate immunity as described, while highlighting the notable exception in macrophage polarization (context-dependent pro-M1 in acute/high-DAMP settings).

**Table 1:** CGRP modulation of innate immunity.

Innate immune cell	Main effect of CGRP	Mechanism/pathway	Context/specific examples	Overall phenotype: pro- or anti-inflammatory?	Key citation(s) from text
Macrophages	Restrains acute inflammation; spatiotemporally regulated polarization	cAMP/PKA pathway (subacute/chronic phases)	Acute phase + high DAMPs → biases toward pro-inflammatory M1 Subacute/chronic phases (e.g., SCI) → shifts to anti-inflammatory, tissue-repairing M2; promotes debris clearance and IL-10 secretion	Context-dependent → Pro-inflammatory (acute/high DAMPs) → Anti-inflammatory (subacute/chronic)	(14, 34–36) Note: Primarily murine mechanistic (SCI models); human receptor expression confirmed
Mast cells	Inhibits degranulation	Not specified in detail	Prevents release of histamine and prostaglandins	Anti-inflammatory	(12) Note: Primarily *in vitro*/human ex vivo; murine confirmation
Neutrophils	Limits recruitment and bactericidal activity	Not specified in detail	Reduces neutrophil influx and killing function	Anti-inflammatory	(37, 38, 41) Note: Primarily murine models; human correlative
Type 2 innate lymphoid cells (ILC2s)	Forms negative feedback loop: suppresses proliferation and cytokine production	Acts via RAMP1 receptor; endogenous CGRP release upon IL-33 stimulation	Strongly inhibits ILC2 proliferation and IL-13 production Prevents runaway mucosal allergic inflammation (e.g., airway); Ramp1 knockout → exacerbated allergic inflammation and eosinophilia	Anti-inflammatory (brake on type 2 inflammation)	(6) Note: Murine knockout + human expression data
Natural killer (NK) cells	Inhibitory effects (direct and indirect)	Increases intracellular cAMP (direct) Indirect via CAFs (RAMP1) in disease models	Early finding: inhibits NK cell functions via cAMP In PDAC: binds RAMP1 on CAFs → halts IL-15 secretion → starves NK cells → promotes tumour survival	Anti-inflammatory/Immunosuppressive (especially in tumour context)	(39, 40) Note: Early human *in vitro* + murine tumour models

## CGRP regulation of adaptive immunity

While CGRP predominantly restrains innate responses in peripheral tissues, its effects on adaptive immunity reveal even greater context-dependence.

Historically, CGRP has been viewed as a Th2-polarizing agent that suppresses Th1 cytokines (63, 64) such as interferon-gamma (IFN-γ) and IL-2, while frequently polarizing cells toward Th2 or Th17 profiles (65–68). Mechanistically, these diverse immunosuppressive effects are primarily driven by the activation of the cAMP and PKA signalling cascades, which inhibit pro-inflammatory transcription factors and induce regulatory networks (20, 25, 26, 41, 52).

Interestingly, emerging evidence suggests it can paradoxically promote Th1 differentiation to clear specific acute viral infections (22). During acute viral infections, such as lymphocytic choriomeningitis virus (LCMV), sensory neurons release CGRP, which binds to RAMP3 on CD4+ T cells, triggering a CREB- Activating Transcription Factor 3 (ATF3) signalling axis. This directly induces the transcription factor Signal Transducer and Activator of Transcription 1 (STAT1), actively promoting Th1 differentiation and rapid viral clearance (22).

In the context of chronic stress, CGRP is a primary driver of cytotoxic T cell exhaustion. Nociceptor-derived CGRP binds to RAMP1 on CD8+ T cells, significantly impairing their cytolytic capacity (8). Recent Clustered Regularly Interspaced Short Palindromic Repeats (CRISPR) screens have identified the epigenetic mechanism behind this: CGRP-RAMP1 signalling upregulates the transcriptional repressor PR Domain Zinc Finger Protein 12 (Prdm12), inducing chromatin remodelling that locks CD8+ T cells into an exhausted phenotype (characterized by high PD-1 and TIM-3 expression) (33). Deletion of Prdm12 in CD8+ T cells reverses this effect, enabling the cells to resist CGRP-induced exhaustion, thereby enhancing antitumour immunity and increasing the production of effector cytokines such as IL-2, IFN-γ, and TNF-α. Furthermore, Prdm12 KO induces epigenetic reprogramming by increasing H3K9me3 (histone H3 lysine 9 trimethylation) deposition at the promoters of specific exhaustion-related genes, including Regulator of G-Protein Signalling 1 (Rgs1), Tribbles Pseudokinase 1 (Trib1), Nuclear Receptor Subfamily 4, Group A, Member 2 (Nr4a2), and Serum- and Glucocorticoid-regulated Kinase 1 (Sgk1), which suppresses these genes and promotes a robust effector phenotype.

Interestingly, adaptive immune cells can also exert retrograde control over these neural signals. A newly discovered neuro-immune circuit shows that tumour-infiltrating B cells actively secrete the endogenous opioid nociceptin (N/OFQ), which binds to OPRL1 receptors on nociceptors, silencing their ability to release CGRP (69). This B cell-mediated silencing of the nerve relieves CGRP-induced T cell suppression, effectively awakening tumour immunosurveillance. CGRP acts as a potent negative regulator of dendritic cells (DCs).

CGRP acts as a potent negative regulator of dendritic cells (DCs).CGRP actively suppresses the maturation and antigen-presenting capabilities of DCs (9). In medullary thyroid cancer (MTC), high levels of tumour-derived CGRP inhibit DC maturation by activating cAMP pathways that prevent the normal down-regulation of Krüppel Like Factor 2 (KLF2) (9). This locks DCs in a dysfunctional state with significantly reduced co-stimulatory molecules (CD80, CD86, HLA-DR), fundamentally impairing T cell priming (9). Beyond their classical roles in maturation and antigen presentation, DC show considerable heterogeneity and functional specialization that plays a key role in shaping immune regulation. This is particularly evident in plasmacytoid DCs (pDCs), which are specialized for the rapid production of type I interferons upon sensing viral nucleic acids via endosomal Toll-like receptor TLR7/9 receptors. However, these responses are tightly controlled by intracellular pathways to prevent harmful overactivation.

Recent studies have provided valuable insights into these regulatory mechanisms. Reizis (2019) offered a comprehensive review of pDC transcriptional programming, emphasizing the central role of TCF4/E2–2 in their development and the Myeloid differentiation primary response 88-Interferon regulatory factor 7 (MyD88-IRF7) pathway that drives robust IFN production while maintaining a balance between antiviral defence and immune tolerance (70). Ngo et al. (2024) highlighted the context-specific functions of pDCs, including their strong IFN responses and relative resistance to certain viral infections in different tissues (71). Hornero et al. (2025) described how TNF and type I IFN crosstalk dynamically controls pDC fate, with TNF favouring differentiation into conventional DC-like cells and IFN-I helping preserve the pDC phenotype during inflammation (72). In addition, Schloer et al. (2025) identified the X-linked factor DEAD-box helicase 3 (DDX3) as a key driver of female-biased IFN-α production through enhanced IRF7 activity upon TLR stimulation (73).

These findings nicely complement our discussion of CGRP as a context-dependent neuroimmune modulator. They suggest that, similar to these intracellular and local signals, CGRP may help fine-tune DC functions and influence adaptive immune responses. Incorporating this broader perspective strengthens the manuscript’s framework of microenvironment-driven immunomodulation.

Of note, the promotion of Th1 responses during acute viral infection (via RAMP3) and Prdm12-mediated CD8^+^ T cell exhaustion were demonstrated in murine LCMV and tumour models; human tumour microenvironment studies provide strong correlative support for CGRP-driven immunosuppression (8, 9, 22, 33).

**Figure 1:**
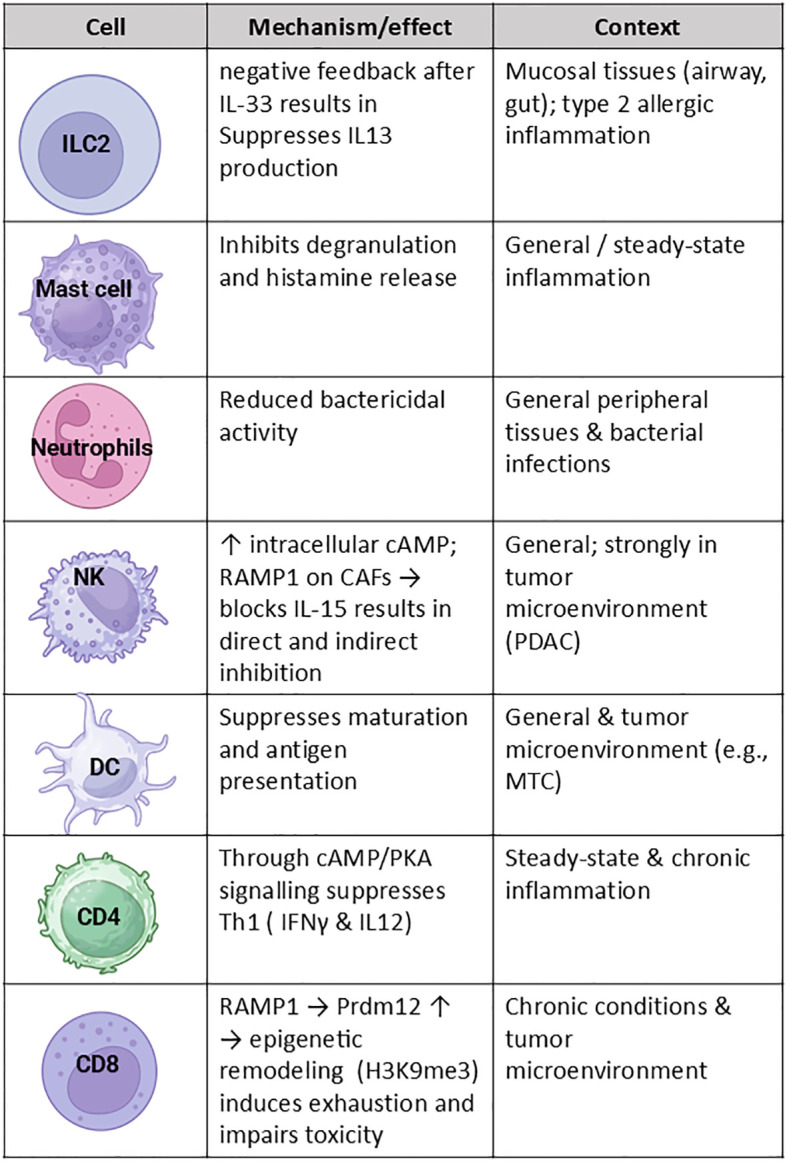
CGRP plays anti-inflammatory/tolerogenic roles. Figure adapted from a template created in BioRender.com.

**Figure 2:**
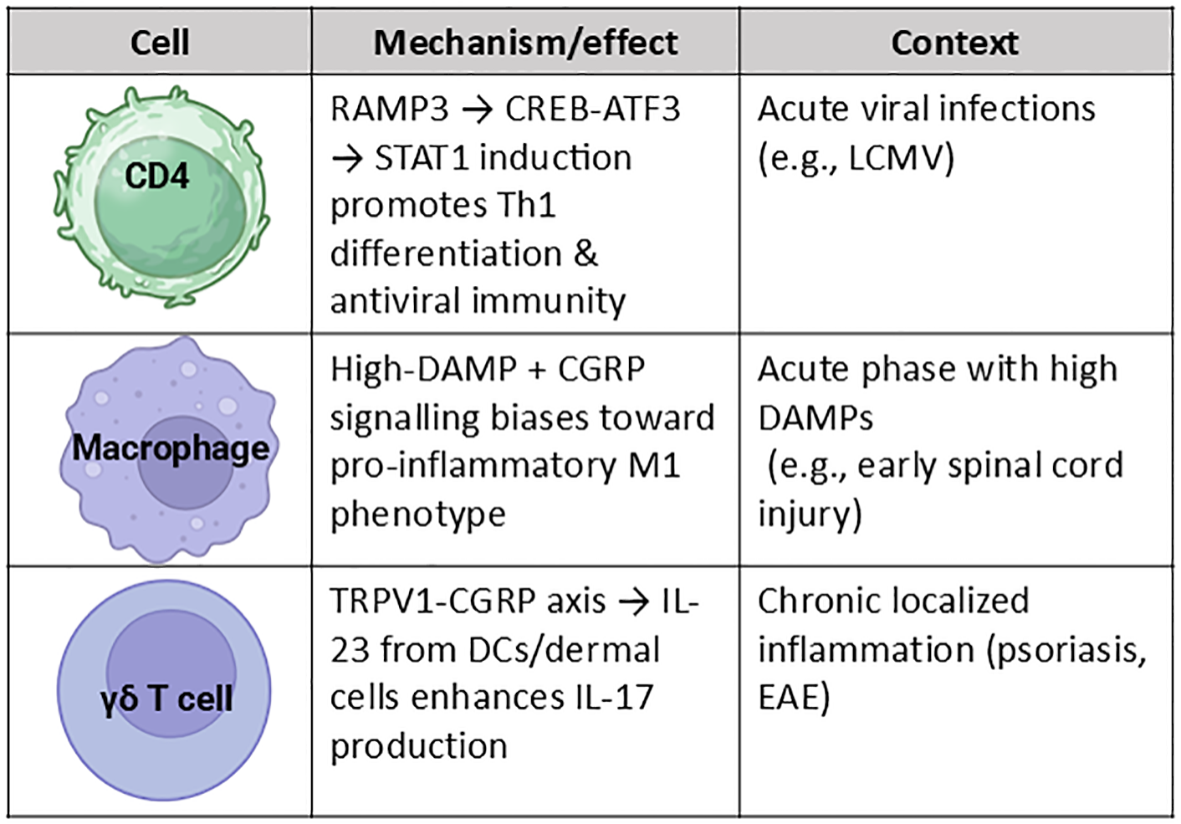
CGRP plays pro-inflammatory roles. Figure adapted from a template created in BioRender.com.

CGRP context-dependant roles on immune cells of both innate and adaptive are illustrated in **Figures 1**, **2**.

**Table 2** highlights the intense context-dependence emphasized in the paragraph: CGRP is mostly immunosuppressive (anti-inflammatory) in chronic settings or steady-state (suppressing Th1, driving Th2/Th17, exhausting CD8+ T cells), but pro-inflammatory in acute viral challenges (promoting Th1 via RAMP3.

**Table 2:** CGRP regulation of adaptive immunity.

Adaptive immune cell/subset	Main effect of CGRP	Mechanism/pathway	Context/specific examples	Overall phenotype: pro- or anti-inflammatory?	Key citation(s) from text
CD4+ T cells (general/historical view)	Suppresses Th1 cytokines; polarizes toward Th2 or Th17 profiles	cAMP/PKA signalling cascades; inhibits pro-inflammatory transcription factors; induces regulatory networks	Generally suppresses IFN-γ and IL-2 production; promotes Th2/Th17 skewing	Anti-inflammatory/Immunosuppressive (Th1-suppressive, Th2/Th17-promoting)	(63) (64) (65) (66) (67) (41, 52) (25, 26) (20) Note: Primarily murine + *in vitro*; human correlative
CD4+ T cells (acute viral infection exception)	Paradoxically promotes Th1 differentiation and cytokine production	Binds RAMP3 on CD4+ T cells → triggers CREB-ATF3 axis → induces STAT1 transcription factor	Acute viral infections (e.g., LCMV); sensory neuron-derived CGRP drives rapid Th1 response and viral clearance	Pro-inflammatory (Th1-promoting, enhances antiviral immunity)	(22) Note: Murine LCMV model; limited human support
CD8+ T cells (cytotoxic)	Drives exhaustion; impairs cytolytic capacity	Binds RAMP1 → upregulates transcriptional repressor Prdm12 → induces chromatin remodelling (H3K9me3 deposition at exhaustion-gene promoters)	Chronic stress or tumour microenvironment; locks into exhausted phenotype (high PD-1, TIM-3); reduces effector cytokines (IL-2, IFN-γ, TNF-α)	Anti-inflammatory/Immunosuppressive (exhaustion-promoting, impairs antitumour/cytotoxic function)	(8) (33) Note: Murine tumour models + human correlative TME data
CD8+ T cells (with Prdm12 modulation)	Prdm12 deletion reverses exhaustion; enhances effector function	Prdm12 KO → reduces Ramp1 expression; increases H3K9me3 at promoters of exhaustion genes (Rgs1, Trib1, Nr4a2, Sgk1); augments effector programs	Experimental (CRISPR screens); restores cytolytic capacity, cytokine production, and antitumour immunity despite CGRP exposure	Anti-exhaustion/Pro-effector (rescues from CGRP suppression)	(33) Note: Murine CRISPR; human tumour relevance
Tumour-infiltrating B cells (retrograde control)	Secrete nociceptin (N/OFQ) to silence CGRP release from nociceptors	Binds OPRL1 on nociceptors → inhibits CGRP release	Tumour microenvironment; relieves CGRP-induced T cell suppression → awakens immunosurveillance	Pro-inflammatory/Immunostimulatory (counteracts CGRP immunosuppression)	(69) Note: Murine tumour model
Dendritic cells (DCs)	Potent negative regulator: suppresses maturation and antigen-presenting capabilities	Activates cAMP pathways; prevents down-regulation of KLF2	Locks DCs in dysfunctional state with reduced co-stimulatory molecules (CD80, CD86, HLA-DR); impairs T cell priming Particularly in medullary thyroid cancer (MTC) with high tumour-derived CGRP	Anti-inflammatory/Immunosuppressive	(9) Note: Human MTC tumour data + murine mechanisms

## The pathological dichotomy: autoimmunity, inflammation, and infection

CGRP treads a fine line between protective homeostasis and pathogenic inflammation. Systemically, CGRP acts as a vasoprotective and anti-inflammatory agent. During severe systemic stress, such as catheter-related bloodstream infections (BSIs) or sepsis, serum CGRP levels drop significantly in infected patients (74, 75). This depletion reflects a breakdown in neuro-immune surveillance, resulting in unchecked vascular permeability and immune dysregulation that facilitates further microbial invasion (74, 75).

Conversely, the chronic activation of the TRPV1-CGRP axis actively drives localized inflammatory diseases. In imiquimod (IMQ)-induced psoriasis models, the activation of nociceptive sensory neurons leads to a massive surge of CGRP, which acts on dermal cells to drive the initial production of IL-23 (76). This, in turn, fuels the pathogenic release of IL-17A from gamma-delta T cells (76). Denervating these sensory fibres with resiniferatoxin (RTX) completely abolishes CGRP release and halts the psoriatic cascade (76). CGRP exacerbates experimental autoimmune encephalomyelitis by promoting Th17 functions (77). In addition, in psoriasis and *C. elegans* infection models, CGRP stimulates DCs to produce IL-23 thereby facilitating the inappropriate activation of IL-17-producing Gamma delta T cells (gdT) (11, 78). Finally, in a model of optogenetic activation to TRPV1+ nociceptors, induction of type 17 inflammation requires the vesicle release of CGRP (79). **Table 3** captures the paradoxical duality described: CGRP is protective/anti-inflammatory systemically under normal or resolved conditions (vasoprotective, maintains homeostasis), but its depletion in severe systemic stress (e.g., sepsis) loses this protection and worsens outcomes. Conversely, chronic/aberrant overactivation (especially via TRPV1+ nociceptors) drives pro-inflammatory pathology in localized diseases like psoriasis and Experimental autoimmune encephalomyelitis (EAE), often via IL-23/IL-17/type 17 pathways.

**Table 3:** Pathological dichotomy of CGRP in inflammation.

Context/setting	Main effect of CGRP	Mechanism/pathway/key examples	Overall phenotype: pro- or anti-inflammatory?	Key citation(s) from text
Systemic (protective homeostasis)	Vasoprotective and anti-inflammatory agent; maintains neuro-immune surveillance	High/normal levels support vascular integrity and balanced immunity; depletion leads to breakdown	Anti-inflammatory/Protective (systemic stress resolution)	(74, 75) Note: Human clinical (migraine/sepsis cohorts) + murine
Systemic severe stress (e.g., sepsis, BSIs)	Significant drop in serum CGRP levels in infected patients	Depletion reflects impaired neuro-immune surveillance → unchecked vascular permeability + immune dysregulation → facilitates microbial invasion	Loss of anti-inflammatory protection (depletion promotes pathogenic inflammation)	(74) Note Human clinical/observational
Localized chronic/aberrant activation (pathogenic inflammation)	Drives localized inflammatory diseases via chronic TRPV1-CGRP axis activation	Nociceptive sensory neuron surge of CGRP → acts on local cells (e.g., dermal cells, DCs) to promote pro-inflammatory cascades	Pro-inflammatory/Pathogenic (exacerbates disease)	(77–79) Note: Strong murine models + human correlative
Psoriasis (IMQ-induced model)	Massive CGRP surge drives initial IL-23 production; fuels IL-17A from γδ T cells	CGRP from activated TRPV1+ nociceptors → stimulates dermal cells/DCs → IL-23 → pathogenic IL-17A release; RTX denervation abolishes CGRP and halts cascade	Pro-inflammatory (initiates/maintains psoriatic cascade)	(76) Note: Primarily murine mechanistic; human correlative
Psoriasis & C. elegans infection models	Stimulates DCs to produce IL-23; facilitates inappropriate activation of IL-17-producing γδ T cells	CGRP acts on DCs → IL-23 production → drives γδ T cell IL-17 release	Pro-inflammatory (Th17/type 17 skewing)	(11, 78) Note: Murine models + human correlative
Experimental autoimmune encephalomyelitis (EAE)	Exacerbates disease by promoting Th17 functions	CGRP enhances Th17 cell activity and IL-17 expression	Pro-inflammatory (worsens autoimmune neuroinflammation)	(77) Note: Primarily murine
Optogenetic TRPV1+ nociceptor activation model	Required for induction of type 17 inflammation	Vesicle release of CGRP from activated TRPV1+ nociceptors is essential for type 17 response	Pro-inflammatory (necessary for type 17 inflammation)	(79) Note: Murine mechanistic

## CGRP in the tumour microenvironment (cancer neuroscience)

Building upon CGRP’s established role as a key modulator in the resolution phase of inflammation, its functions extend logically into the tumour microenvironment, where it continues to act as a potent “double agent.” In cancer, nerve-derived CGRP released by tumour-recruited sensory fibres drives immunosuppression by impairing cytotoxic T-cell function, dendritic cell maturation, and NK-cell activity, thereby converting a physiologic resolution program into pathological immune evasion (9, 17, 80). Recent studies further demonstrate that tumours actively hijack this neuro-immune axis; for instance, melanoma cells stimulate nociceptor neurons to increase CGRP release, which directly suppresses anti-tumour immunity, while cancer cells can also co-opt distant inter-organ circuits via Slit Guidance Ligand 2 (SLIT2) to trigger CGRP-mediated immunosuppression in tumour-draining lymph nodes (7, 81). This section thus examines CGRP-mediated neuro-immune crosstalk in oncology as a direct extension of its broader immunomodulatory repertoire.

The rapidly expanding field of “cancer neuroscience” has revealed that solid tumours actively hijack CGRP-expressing sensory nerves to establish a highly permissive, immunosuppressed microenvironment (50, 82, 83). Tumours actively recruit and remodel sensory nerves to establish an immunologically “cold” and permissive niche (7–9, 60, 84, 85). Tumours release mediators such as neuronal growth factor (NGF) and adenosine. NGF will induce the sprouting of CGRP-expressing sensory nerves into the tumour bed (50, 60) while the latter engages A2A receptors on nociceptors—to also trigger the robust release of CGRP into the tumour bed (60, 86). Once released, CGRP bridges the gap between tumour-associated pain and immune evasion (17) Subsequently, tumours exploit nerve-derived CGRP to directly induce exhaustion in cytotoxic CD8+ T cells, lock DCs in an immature state, and suppress the anti-tumour activity of NK cells, thereby evading immune clearance and accelerating malignant progression (9, 17, 80). Indeed, solid tumours undergo “neural addiction,” actively co-opting CGRP networks. CGRP directly binds to CLR/RAMP1 on cancer cells, driving survival and proliferation pathways (50). CGRP released by cancer cells acts in a RAMP1-dependent manner to enhance tumour cell growth, proliferation, metabolism, and migration. In gastric cancer, this axis activates the Retinoblastoma/E2 promoter-binding Factor (Rb/E2F) pathway; in bone-metastatic prostate and breast cancers, it heavily activates the p38/HSP27 cascade (27); and in colorectal cancer, it directly accelerates tumour cell growth (83). Furthermore, CGRP is found to promote GI tumour growth through the upregulation RAMP1 which negatively correlated with survival rates of both primary colorectal cancer and gastric cancer (83).

In terms of CGRP prominent role in pain perception and its association with the progression of cancer, CGRP release intimately links cancer-associated pain with profound immunosuppression. In head and neck squamous cell carcinoma (HNSCC) and oral squamous cell carcinoma (OSCC), elevated densities of CGRP+ nerves strongly correlate with severe patient-reported oral pain and a simultaneous depletion of tumour-infiltrating CD8+ T cells (17). Interestingly, The tumour evokes pain to stimulate nociceptors, which reciprocate by releasing CGRP to exhaust local T cells and protect the tumour (8). Furthermore, CGRP signalling actively remodels the extracellular matrix by upregulating matrix metalloproteinases (e.g., MMP-2, MMP-9), facilitating aggressive perineural invasion (PNI) along white-matter tracts and cranial nerves in glioblastomas and head and neck cancers (8, 87–89).

Interestingly, neuro-immune signalling is not entirely unidirectional; recent findings show that tumour cells produced CGRP can drive autocrine proliferation and angiogenesis (VEGFA) in gastrointestinal cancers (83). Furthermore, CGRP and its sister neuropeptides (Substance P, Pituitary Adenylate Cyclase-Activating Polypeptide (PACAP)) actively sculpt the extracellular matrix to forge immune-excluded pathways for PNI. By upregulating MMP-2, MMP-9, CGRP and Substance P allow glioblastoma (GBM) and HNSCC cells to seamlessly tunnel along myelinated white-matter tracts and cranial nerves, escaping localized immune attacks (76, 86). In conclusion, this pro-tumour activity in cancer represents the pathological extreme of CGRP’s resolution-phase immunosuppressive functions when hijacked by tumours.

**Table 4** captures the “neural addiction” concept: Tumours actively co-opt CGRP networks for growth, pain, and immunosuppression, turning a normal neuro-immune signal into a pathological driver.

**Table 4:** CGRP in cancer neuroscience and tumour microenvironment.

Aspect/process	Main role/effect of CGRP	Mechanism/pathway/key examples	Overall phenotype: pro- or anti-tumour?	Key citation(s) from text
Tumour-nerve recruitment & remodelling	Tumours actively recruit and remodel CGRP-expressing sensory nerves to create an immunologically “cold” niche	Tumours secrete NGF → nerve sprouting into tumour bed; adenosine activates A2A receptors on nociceptors → robust CGRP release	Pro-tumour (establishes permissive immunosuppressive TME)	(21, 40, 60–64) Note: Murine + human tumour innervation studies
Immune evasion in TME	Suppresses key anti-tumour effectors: CD8^+^ T cell exhaustion, DC maturation arrest, NK cell suppression	Nerve-derived CGRP → RAMP1 signalling → Prdm12-mediated epigenetic exhaustion of CD8^+^ T cells; cAMP pathway locks DCs in immature state (↓CD80/CD86/HLA-DR); indirect NK suppression via CAF-derived IL-15 blockade (PDAC)	Pro-tumour (robust immunosuppression & immune evasion)	(22, 23, 32, 40, 57, 58) Note: Murine mechanistic + strong human tumour correlative
Direct effects on cancer cells	Promotes tumour cell survival, proliferation, metabolism, migration, and angiogenesis	CGRP binds CLR/RAMP1 on tumour cells → Rb/E2F (gastric), p38/HSP27 (bone-metastatic prostate/breast), general growth acceleration (colorectal); tumour-derived CGRP autocrine loop; ↑VEGFA	Pro-tumour (direct growth promotion & autocrine stimulation)	(17, 61, 62) Note: Human + murine GI/prostate data
Cancer-associated pain & immunosuppression link	Links tumour-evoked pain to immune evasion; elevated CGRP^+^ nerve density correlates with severe pain and depleted TILs	Tumour stimulates nociceptors → CGRP release → exhausts local CD8^+^ T cells; high CGRP^+^ innervation in HNSCC/OSCC	Pro-tumour (pain actively drives progression via immunosuppression)	(22, 57) Note: Strong human OSCC/HNSCC patient data
Perineural invasion (PNI) & ECM remodelling	Facilitates aggressive perineural invasion and immune-excluded pathways	CGRP (with SP/PACAP) upregulates MMP-2/MMP-9 → ECM remodelling → tunnelling along nerves/white-matter tracts (GBM, HNSCC)	Pro-tumour (promotes invasion & escape from immune surveillance)	(22, 53, 65–68) Note: Murine + human histopathological
Neural addiction & overall tumour progression	Tumours become “addicted” to CGRP signalling for growth, pain, and immune protection	Pathological hijacking of resolution-phase immunosuppressive functions of CGRP; RAMP1 upregulation negatively correlates with survival in GI cancers	Strongly Pro-tumour (converts physiologic resolution program into pathological driver)	(58, 60–62) Note: Human survival correlation + murine

## Translational therapeutics and future directions

The systemic integration of CGRP in pain, inflammation, and oncology presents a highly lucrative target for pharmacological intervention. FDA-approved migraine therapies—such as small-molecule CGRP receptor antagonists (gepants like rimegepant and olcegepant) and monoclonal antibodies (Eptinezumab, Erenumab, Fremanezumab, Galcanezumab)—are primed for oncological repurposing (9, 17, 23, 60). Because endosomal signalling is critical for sustained pain and inflammatory signalling, nanoparticle-encapsulated olcegepant has been successfully engineered to traffic directly into early endosomes, providing vastly superior, long-lasting relief from OSCC-induced bone pain compared to other drugs (23).

Looking forward, disrupting the neuro-immune axis via CGRP blockade offers immense synergistic potential. Antagonizing CGRP could be paired with immune checkpoint blockade (e.g., anti-PD-1) to rescue CD8+ T cells from epigenetically locked exhaustion (8, 33). Additionally, because sensory neurons can induce docetaxel chemoresistance in triple-negative breast cancer, combined CGRP/Substance P blockade may re-sensitize resistant tumours to standard chemotherapies (82). Preclinical administration of these agents successfully slows tumour growth, mitigates bone and oral cancer pain, and restores the infiltration and cytolytic functions of CD8+ T cells and NK cells (17, 27, 60). Furthermore, combining CGRP blockade with established treatments like radiotherapy or immune checkpoint inhibitors i.e. anti-PD-1 synergistically overcomes neural-driven therapeutic resistance (8, 90, 91). Therapeutic implementation must be carefully calibrated; because CGRP is essential for maintaining mucosal barriers and resolving acute inflammation, chronic systemic antagonism carries the risk of impairing normal tissue healing or exacerbating subclinical infections, necessitating targeted, context-aware delivery (24, 80, 86). This concern has been emerging in other fields, in particular migraine management where anti-CGRP monoclonal antibodies have are hailed as a breakthrough. Finally, while many synergistic effects with checkpoint inhibitors were shown in murine tumour models, early human correlative data and the established safety profile of anti-CGRP mAbs in migraine patients support translational feasibility (9, 17). Future studies should prioritize human interventional trials.

## Conclusion

**Figure 3:**
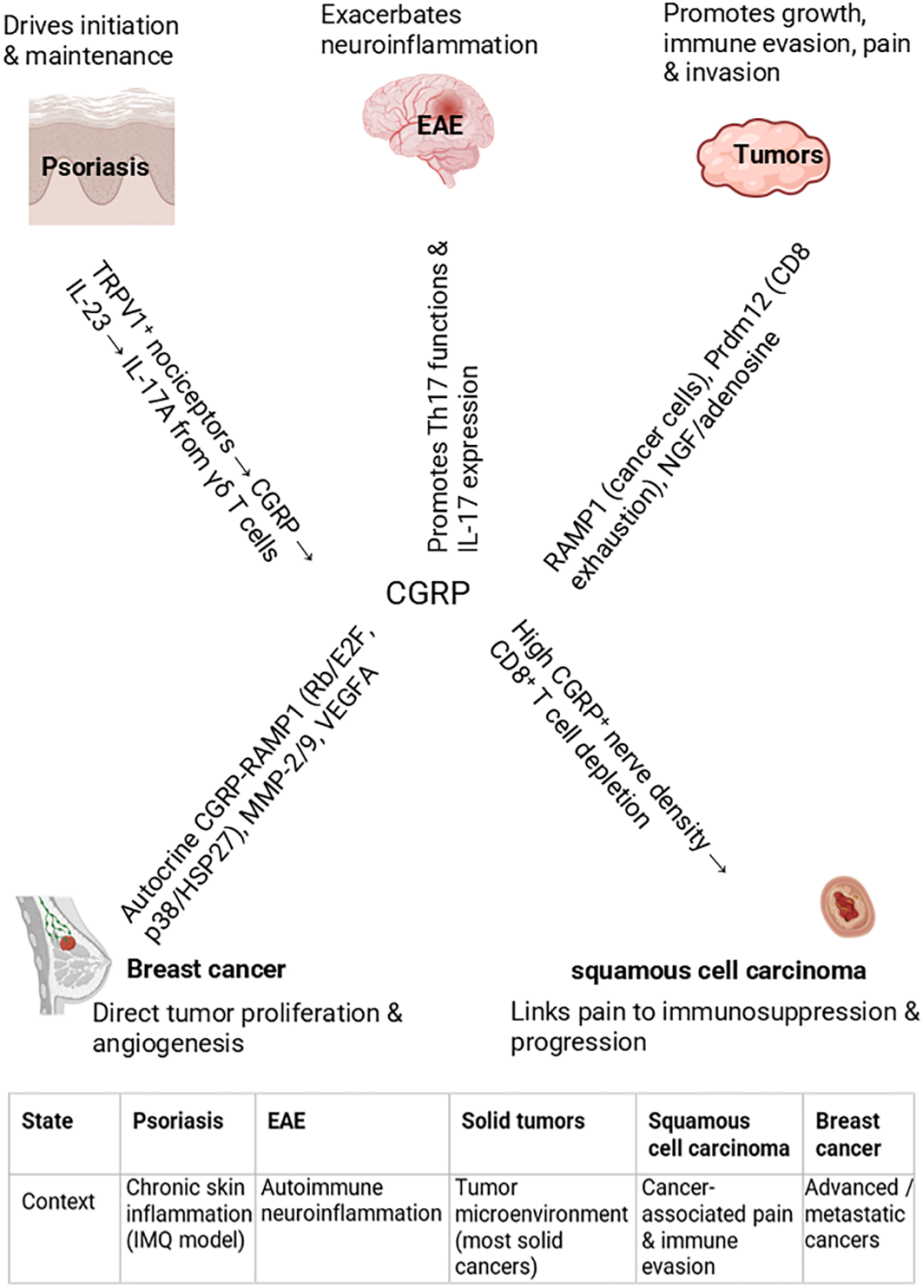
CGRP either promotes or exacerbates certain pathologies. Figure adapted from a template created in BioRender.com.

**Figure 4:**
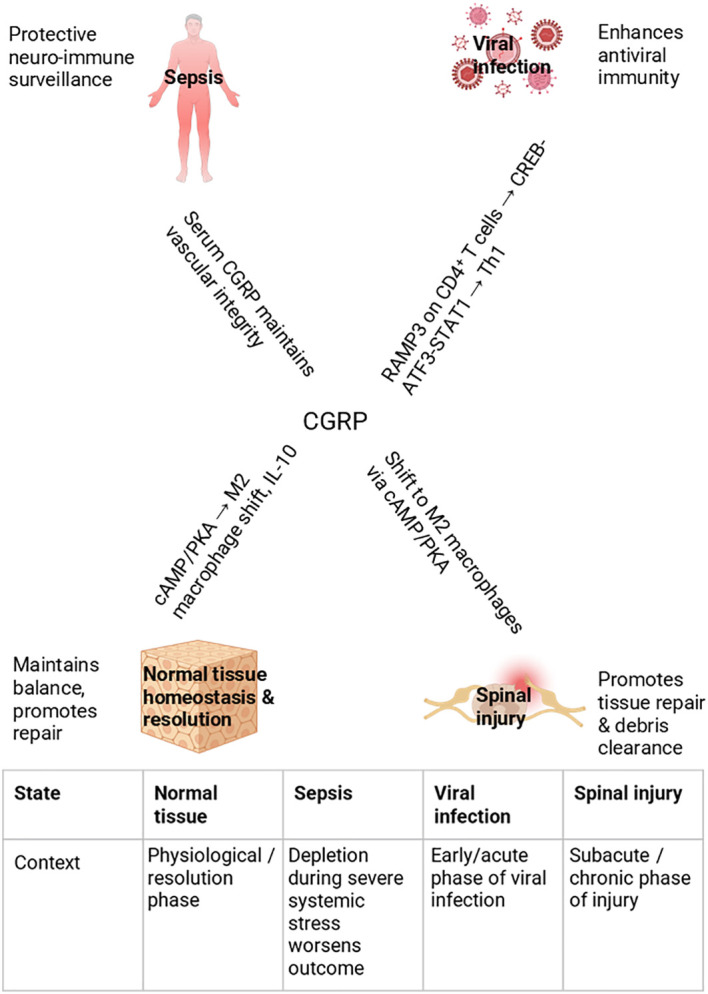
CGRP depletion either protective or worsens certain pathologies. Figure adapted from a template created in BioRender.com.

CGRP stands as a master orchestrator at the neuro-immune interface, seamlessly bridging sensory perception with systemic immune modulation (2, 20, 80). This review highlights the multiple, often contradictory roles, CGRP plays in different pathologies/diseases swinging often between pro-inflammatory destructive roles and anti-inflammatory protective roles (**Figures 3**, **4**). While its basal physiological function is designed to protect tissue integrity by restraining excessive inflammation, solid tumours pathologically hijack this exact nociceptive circuitry to shield themselves from cytotoxic immune clearance and fuel their own growth and invasion (80, 86). The integration of cancer biology and neuroimmunology has definitively shown that tumour-induced pain is not merely a byproduct of disease, but an active driver of tumour progression via CGRP release (17). Moving forward, the targeted disruption of the CGRP/RAMP1 signalling axis—particularly through repurposed migraine therapeutics—offers a paradigm-shifting strategy in oncology: the ability to simultaneously alleviate severe cancer-associated pain while dismantling the immunosuppressive tumour microenvironment (17). To fully realize this potential, future clinical endeavours must prioritize localized delivery systems, endosomally targeted nanoparticle formulations, and biomarker-guided patient stratification to safely untangle this neuro-immune circuitry without compromising systemic homeostasis (23, 76, 80).
